# nf-core/magmap: Map metatranscriptomes to large collections of genomes

**DOI:** 10.1093/bioinformatics/btag501

**Published:** 2026-07-10

**Authors:** Danilo Di Leo, Emelie Nilsson, George Westmeijer, Jarone Pinhassi, Daniel Lundin

**Affiliations:** Centre for Ecology and Evolution in Microbial Model Systems, EEMiS, Linnaeus University, Kalmar SE-39182, Sweden; Centre for Ecology and Evolution in Microbial Model Systems, EEMiS, Linnaeus University, Kalmar SE-39182, Sweden; Department of Chemistry, Umeå University, Umeå SE-90187, Sweden; Centre for Ecology and Evolution in Microbial Model Systems, EEMiS, Linnaeus University, Kalmar SE-39182, Sweden; Centre for Ecology and Evolution in Microbial Model Systems, EEMiS, Linnaeus University, Kalmar SE-39182, Sweden

## Abstract

**Summary:**

The lack of publicly available reference genomes has forced annotation of metatranscriptomes to either use direct alignment of sequence reads to reference databases or de novo assembly. As more and more natural environments are covered by metagenomic surveys, this is rapidly changing. This opens up the possibility of genome-resolved studies of prokaryotic metatranscriptomes by mapping to genomes from public repositories or metagenome-assembled genomes derived from the same environment. Here, we present the nf-core/magmap pipeline that provides a reproducible, easy-to-access, and well-documented workflow for selecting reference genomes, mapping to them, and quantifying features. Genomes can be drawn from public sources or originate from private collections. The pipeline is primarily aimed at prokaryotic communities but can, together with collections of reference mature gene sequences, also be applied to eukaryotes.

**Availability and implementation:**

The nf-core/magmap pipeline is implemented in Nextflow and part of the nf-core collaboration. The pipeline is available at the nf-core website (https://nf-co.re/magmap) and GitHub (https://github.com/nf-core/magmap).

## 1 Introduction

**Figure 1:**
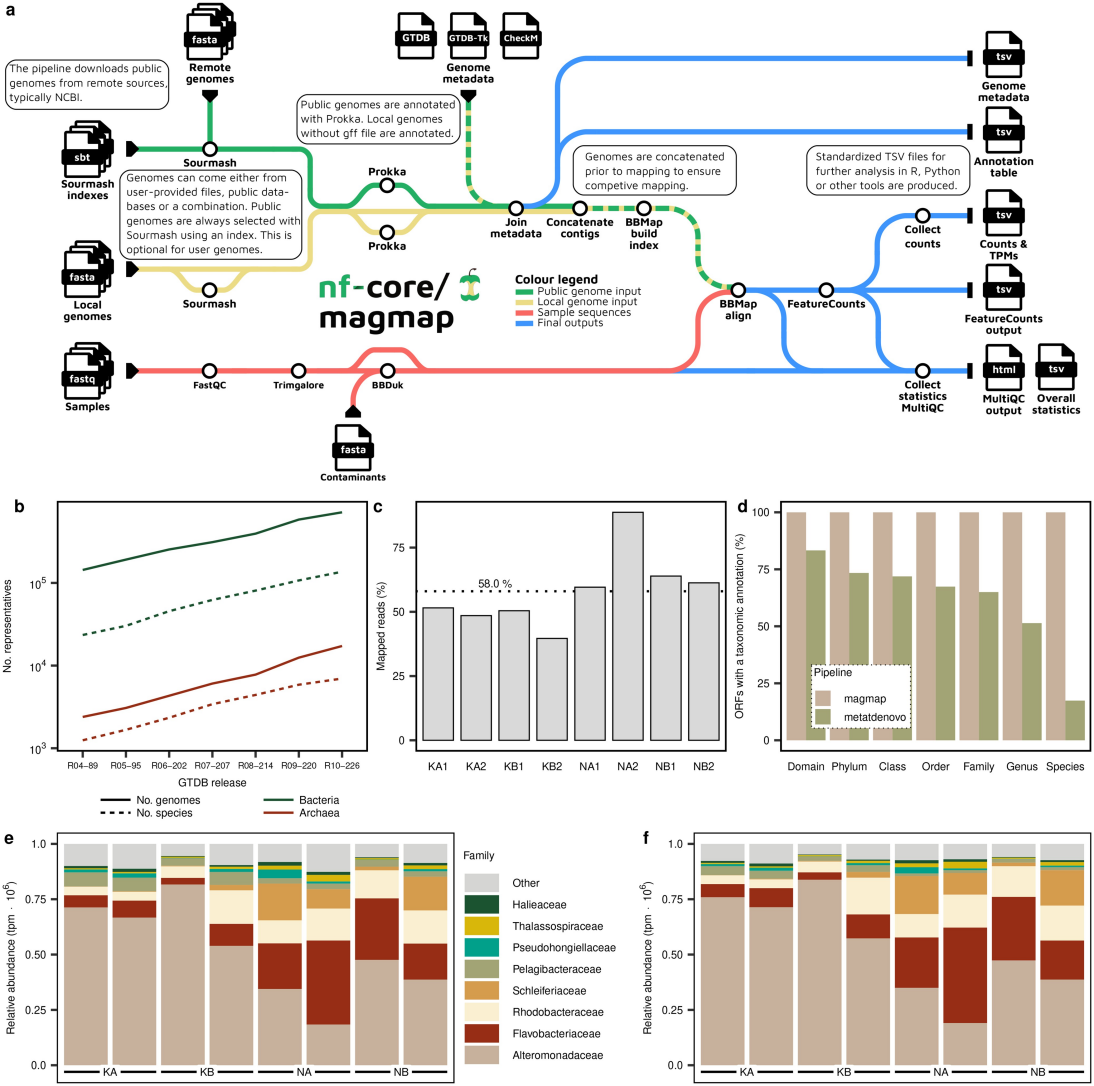
Overview of nf-core/magmap performance. (a) Data flow in nf-core/magmap. (b) Increase in the number of genomes and species in GTDB since the first public release. (c) Percentage of metatranscriptomic reads that mapped to reference genomes in the GTDB release R10-RS226. The dotted line depicts the average mapping percentage (range 45.3–77.0 %, *n* = 8). (d) Taxonomic resolution of ORFs processed with nf-core/magmap and nf-core/metatdenovo. (e) Community structure according to metatranscriptomes processed with nf-core/magmap. Showing the eight most transcriptionally active families while grouping less active taxa as ‘Other’. (f) Community structure according to nf-core/metatdenovo, including the families identified to be highly active in ‘e’.

Annotation of metatranscriptomes has frequently relied on direct annotation of sequencing reads using fast alignment algorithms such as Diamond (Buchfink *et al*. 2015), or de novo assembly of reads into contigs (Anwar *et al*. 2019). In both cases, similarity searches against reference databases such as NCBI’s NR (Sayers *et al*. 2024) or RefSeq (Goldfarb *et al*. 2025) provide functional and taxonomic information. A clear disadvantage of these approaches is that genomic context is lacking and, following from that, taxonomic precision differs between genes. This makes it difficult to study the expression of pathways rather than single genes unless genes of interest are encoded by operons that de novo assembly manages to recover. The differences in taxonomic precision also make it difficult to compare the expression of individual genes at arbitrary taxonomic ranks. Until recently, the lack of reference genomes covering the diversity of many natural environments has hindered the alternative approach–direct mapping of sequencing reads to genomes–and, with that, genome-resolved annotation of metatranscriptomes was not a possibility (Anantharaman *et al*. 2016, Bandla *et al*. 2020). A proposed remedy for this problem is to combine the sequencing of metatranscriptomes with the sequencing of metagenomes (Yan *et al*. 2024). However, the retrieved metagenome-assembled genomes (MAGs) often fail to cover the full diversity of the metatranscriptomes (Yan *et al*. 2024). The fact that many natural environments are now extensively covered by metagenome sequencing, leading to a rapid increase in the number of available genomes (Fig. 1b), is changing the outlook of annotation methods based on mapping to reference genomes. Projects from an increasing number of well-covered natural environments could hence achieve genome-resolution by mapping to public reference genomes as a complement to, or replacement for, simultaneous sequencing of metagenomes. Mapping reads to all the hundreds of thousands of available genomes is clearly not feasible with current methods. But if relevant genomes can be selected with a faster and less resource-hungry algorithm before mapping, mapping becomes more achievable. Methods based on kmers and hashing techniques promise to provide this and are implemented in software such as Sourmash (Pierce *et al*. 2019). Moreover, the authors of Sourmash provide indexes for public genome collections, such as the Genome Taxonomy Database (GTDB) (Parks *et al*. 2022) or NCBI databases (Sayers *et al*. 2024), making it possible to identify sets of genomes that are representative of a given set of metatranscriptomic samples. Whether the identified sets indeed capture the biological diversity present in the metatranscriptomic samples is, of course, dependent on how much sequencing and genome reconstruction effort has been spent on the specific environment.

Here, we introduce a pipeline–nf-core/magmap, implemented in Nextflow (Di Tommaso *et al*. 2017) and part of the nf-core community (Ewels *et al*. 2020)—that makes selection of representative genomes followed by mapping of reads to the selection accessible for researchers. The choice of Nextflow as a software platform for the pipeline, together with incorporation in the nf-core community, makes the pipeline easy to install and run without manual dependency management and portable across different hardware platforms. In addition, this design ensures reproducibility, comprehensive documentation, and a higher likelihood of long-term maintenance compared to pipelines developed by individual labs (Langer *et al*. 2025). The pipeline is primarily aimed at the study of sequences from prokaryotes, but there is nothing in principle hindering its application to eukaryotic data by mapping to reference transcriptomes such as MMETSP (Keeling *et al*. 2014) or other collections of sequences from mature transcripts.

## 2 Results

### 2.1 Software platform

The pipeline is implemented in Nextflow (Di Tommaso *et al*. 2017), a “workflow orchestrator” that provides support for execution on different hardware platforms–e.g. standalone computers, high-performance compute clusters, and cloud compute environments–using the parallelization provided by each technology. A number of different means to provide software to individual tasks are supported by Nextflow, including container technologies, e.g. Docker (Merkel 2014), Singularity (Kurtzer *et al*. 2017), and Conda (Grüning *et al*. 2018). Pipelines can be run from a UNIX-like command prompt or the Seqera platform, a web-based graphical interface (https://seqera.io/platform/). Interrupted pipeline runs can be resumed from the point of the interruption without recalculation of already executed processes.

### 2.2 Algorithm overview

There are two ways to provide genomes to the pipeline (Fig. 1a, green and yellow paths). They can either be provided by the user (“user-provided” below) or come from public sources (“public”); these two approaches can be combined. Public genomes are selected using Sourmash (Pierce *et al*. 2019), a tool that compares kmer distributions between samples and an index of genomes. The same tool can also be used to filter user-provided genomes. After selection, genomes are annotated with Prokka (Seemann 2014) to provide a ‘gff’ file for the quantification step. To make sure that alignment is competitive, i.e. that reads are mapped to the optimal targets, contigs from all genomes are concatenated before creation of a mapping index for BBMap (https://sourceforge.net/projects/bbmap/). Optionally, if the user provides metadata for genomes, e.g. CheckM (Parks *et al*. 2015), CheckM2 (Chklovski *et al*. 2023), or GTDB-Tk (Chaumeil *et al*. 2019) data or GTDB (Parks *et al*. 2022) metadata files, this information is summarized by the pipeline.

In parallel, sample reads are quality controlled with FastQC (https://www.bioinformatics.babraham.ac.uk/projects/fastqc/) and trimmed with TrimGalore (Krueger *et al*. 2023) (Fig. 1a, red path). Before mapping, reads can also be filtered from contaminants such as host or stable RNA sequences using a user-provided fasta file and BBDuk (https://sourceforge.net/projects/bbmap/). This can substantially improve performance by decreasing the number of sequences to map, and can also improve quality since stable RNA molecules can affect the quantification of coding sequences by unspecific mapping. Subsequently, cleaned reads are mapped to the concatenated genomes using BBMap, and features are quantified with featureCounts from the Subread package (Liao *et al*. 2014).

To make the output more accessible for further analysis, the pipeline outputs tab-separated files with consistent naming of fields using custom code in the pipeline (Fig. 1a, blue path). In these files, feature counts are presented along with length-corrected relative abundances, “transcripts per million” (TPM). In addition, MultiQC (Ewels *et al*. 2016) is run to provide overall statistics.

### 2.3 Analysis of real data and a comparison with nf-core/metatdenovo

We mapped metatranscriptomic reads from Bunse *et al*. (2016) to genomes in GTDB (release R10-RS226) using nf-core/magmap, resulting in an average mapping rate of 58.0% (sd 14.7, *n* = 8; Fig. 1c) to a selection of 8576 GTDB species representative genomes. The same metatranscriptomic reads were recently used to describe nf-core/metatdenovo, a pipeline that is based on de novo assembly of reads, with slightly higher mapping rates between 62.3% and 78.8% depending on settings (Di Leo *et al*. 2025). As species-resolved genomes were used as reference with nf-core/magmap, this implied that all of the mapped ORFs were annotated at the species level, in contrast to the output of nf-core/metatdenovo (Fig. 1d). Consequently, the resolution obtained from nf-core/magmap allows one to perform analysis at any preferred taxonomic rank (e.g. family or genus) without the loss of ORFs that were only assigned to higher taxa, as is the case with de novo assembled data. It also means that the user can perform analyses of genomic co-expression of genes. Focusing on community structure revealed that the eight most transcriptionally active families from the communities based on nf-core/magmap were responsible for 91.1% of the TPM. The same eight families were responsible for 93.0% of the TPM in the communities based on nf-core/metatdenovo, after adjusting the TPM for nf-core/metatdenovo to only consider ORFs with a taxonomic annotation at the family level (Fig. 1e and f). Overall, the community structure was strikingly similar between the two pipelines, suggesting that mapping to reference genomes fetched from public repositories is a valid alternative to de novo assembly when MAGs from the environment are not available or cover diversity poorly, at least in environments well-covered by public genomes.

## 3 Methods

### 3.1 Dataset preparation

To demonstrate how nf-core/magmap performs on real data, we ran version 1.0.0 of the pipeline on data published in Bunse *et al*. (2016). Reads were downloaded from the European Nucleotide Archive with project identifier PRJEB10237. We used the Sourmash (Pierce *et al*. 2019) indexes for GTDB (Parks *et al*. 2018) species-representative genomes release R10-RS226 provided by the Sourmash authors (https://sourmash.readthedocs.io/en/latest/databases.html) to identify genomes to map to and GTDB metadata files to provide genome information.

### 3.2 Code availability

The pipeline code is available at https://github.com/nf-core/magmap. The R code used to produce Fig. 1 is available at https://github.com/LNUc-EEMiS/magmap-manuscript.

## Data Availability

The pipeline code is available at https://github.com/nf-core/magmap. The R code used to produce Fig. 1 is available at https://github.com/LNUc-EEMiS/magmap-manuscript. Sequence data for the analysis is available at the European Nucleotide Archive: http://www.ebi.ac.uk/ena/data/view/PRJEB10237.
